# Next Generation Sequencing of urine exfoliated cells: an approach of prostate cancer microRNAs research

**DOI:** 10.1038/s41598-018-24236-y

**Published:** 2018-05-08

**Authors:** Gabriella Guelfi, Giovanni Cochetti, Valentina Stefanetti, Danilo Zampini, Silvana Diverio, Andrea Boni, Ettore Mearini

**Affiliations:** 1 Department of Veterinary Medicine, University of Perugia, Via S. Costanzo n.4, 06126 Perugia, PG Italy; 2 Department of Surgical and Biomedical Sciences, Section of Urological, Andrological and Minimally invasive techniques, University of Perugia, Piazza Lucio Severi 1, 06132 Perugia, PG Italy

## Abstract

There is emerging evidence that microRNAs (miRNAs) dysregulation is involved in the genesis and the progression of Prostate Cancer (PCa), thus potentially increasing their use in urological clinical practice. This is the first pilot study which utilizes Illumina Deep Sequencing to examine the entire miRNAs spectrum existent in urine exfoliated prostate cells (UEPCs) of PCa patients. A total of 11 male patients with histological diagnosis of PCa were enrolled in the present study. First-catch urine (30 mL) was collected following a prostate massage. Total RNA was extracted from urine and sequenced using an HiSeq2500 System (Illumina). QPCR assay was used to validate the highest NGS results in PCA patients and in age-matched, caucasian men. Remarkably, PCA *let-7* family was down-regulated (P < 0.01), compared to the controls. The results of our study support the notion of a relatively high diagnostic value of miRNA family for PCa detection, especially in the *let-7* family. The present research confirmed the potential use of miRNAs as non-invasive biomarkers in the diagnosis of PCa, potentially reducing the invasiveness of actual clinical strategy.

## Introduction

With the introduction of serum Prostate-Specific Antigen (PSA) test, the incidence of PCa has increased, leading to an increased number of unnecessary biopsies and the diagnosis of clinically insignificant tumors that were not life threatening. PSA has several limitations because of its well-known non-cancer specificity, with raising levels for many other urological conditions such as infections, inflammation or benign prostatic hyperplasia, resulting in high false-positive rates^1^. Prostate biopsy is currently the only way to definitively identify the presence of PCa following anomalous PSA and/or DRE^2^. One of the problems of prostate biopsy is the hitch in tissue sampling follow-on a sensitivity insufficiency (false negatives). Additionally, it should be underlined that prostate biopsy is not only a highly invasive diagnostic tool, but even that patients may suffer from several complications, such as infection, potentially leading to discomfort until up to one month after the procedure^3^. However, prostate biopsy constitutes the gold standard for prostate cancer diagnosis and is the starting point for preoperative pathologic grading and cancer volume estimation. The introduction of multiparametric Magnetic Resonance Imaging (mp-MRI) has improved PCa detection rate, allowing the execution of fusion MRI/ultrasound prostate biopsy. Histological grading which predicts prognosis and helps guide the therapy method is the Gleason score (GS).

It has been universally accepted that one of the main goals in PCa diagnosis and treatment is to detect cancer at an early stage, and to predict which patients will harbor intermediate and high-risk cancer, and thus initiate immediate medical treatment^4^. If diagnosed at an early stage, PCa can be successfully treated with different therapeutic strategies, such as active surveillance, hence delaying or avoiding radical treatment. Rapid and growing advances in whole genome sequencing allows to focus on molecular marker tracking as well as coding and non-coding RNA, revealing different sensitivity and specificity for PCa detection in urine^5,6^. Notably, non-coding RNA biomarkers are microRNAs (miRNAs) that constitute an evolutionarily conserved class of small RNA molecules. MiRNAs comprise one of the more abundant classes of gene regulatory molecules in multicellular organisms and likely influence the output of many protein-coding genes. MiRNAs regulate post-transcriptional gene-expression mainly by binding to the 3′ untranslated region (UTR) of their target messenger RNA (mRNA)^7^ resulting in protein synthesis inhibition^8,9^. MiRNAs play a fundamental role in the regulation of biological functions^10,11^, and there is an emerging evidence that their dysregulation is involved in the genesis and the progression of PCa.

The first human microRNA ever identified, lethal-7 (let-7), is known to be intimately involved in the division and differentiation of stem cells. It has been observed that the expression of let-7 family members is downregulated in many cancer types during tumor progression promoting cell cycle progression. A number of studies revealed in cancer patients significant let-7 family downregulation in renal cell, lung, bladder, colon, gastric, hepatocellular, breast, uterine and ovarian cancer^12^.

It has been demonstrated that miRNAs are detectable in urine of PCa patients collected after DRE (digital rectal examination)^13,14^. As demonstrated in our last study quantitative real-time PCR (qPCR) miRNA analysis could be considered a non-invasive promising tool for diagnostic testing on PCa, and on its prognostic status^15^.

Rapid advancement in miRNAs profiling technologies has made it possible to study microRNAs expression profiles through Next Generation Sequencing (NGS), which provides several advantages respect to methodologies previously employed^16^. NGS showed the largest dynamic range of detection and the greatest detection sensitivity^17,18^; representing a unique tool to investigate the transcriptome complexity at an incredibly high level of resolution^19^.

In our study, the primary research aim was to examine the entire miRNome profiling of UEPCs in PCa patients after DRE, by the NGS approach. Consequently, the highest predicted selection of miRNAs was validated by using qPCR analysis and compared with age-matched healthy control subjects.

## Material and Methods

### Patient selection

Eleven patients with clinical diagnosis of PCa and ages ranging from 51 to 68 years were included in NGS study. In qPCR assay 11 healthy control age-matched caucasian subjects (C) were enrolled, in addition to NGS patients. All samples (PCa and C) were collected for clinical diagnostic tests, after the prostatic massage, at the Hospital of Santa Maria della Misericordia (Perugia, Italy) in the department of Urological, Andrological and Minimally Invasive Techniques. Collection of patient samples has been performed according to national legislation concerning ethical requirements. Use of these samples has been approved by medical ethics committee on 7th February 2013 (protocol n. 2012-11 047R) and the CEAS Committee of Umbria Region on 21 February 2013 (CEAS Register n° 12 2085/13). Written informed consents were obtained from all study subjects and the samples were analyzed anonymously. This research is a retrospective study using archival sample. All oncological patients had a biopsy-proven diagnosis of PCa and underwent robotic radical prostatectomy within 1 month from clinical evaluation. Before biopsy, all patients underwent mp-MRI allowing us to perform a cognitive fusion biopsy technique, with at least 14 cores (median:18, ranging from 16 to 22). All control patients were enrolled after exclusion of any pathological urological conditions. Exclusion criteria were age >75 years, the presence of any other cancer type, bladder urinary lithiasis, urinary infection, previous therapy concerning prostate, as any endoscopic procedures or pharmacological treatment with 5-aromatase inhibitors or alpha-lithics, and presence of family history of PCa. Clinical parameters of subjects enrolled in the study are provided in Table 1.

**Table 1:** Patients’ clinical parameters.

Overall patients	PCa (n. 11)		Healthy (n. 11)	
	Mean	Range	Mean	Range
Age (yy)	61	(51–67)	62	(50–68)
PSA (ng/mL)	4.1*	(1–8)	1.1	(0.3–2)
Volume of Prostate (g)	40	(22–58)	43	(20–60)
Body Mass Index (kg/m^2^)	25.2	(21–30)	26.1	(22–31)
ECOG** performance status	1	(0–3)	1	(0–3)
		**Clinical n (%)**	**Pathological n (%)**	
Stage	T1	8 (72.7)	0	
	T2 (a–c)	3 (17.3)	11 (100)	
Gleason Score	3 + 3	6 (54.5)	4 (36.3)	
	3 + 4	3 (27.3)	2 (18.2)	
	4 + 3	2 (18.2)	3 (27.3)	
	4 + 4	0	1 (9.1)	
	4 + 5	0	1 (9.1)	

*PSA value at diagnosis. **ECOG (Eastern Cooperative Oncology Group).

### Urine collection and total RNA extraction

First-catch urine (roughly 30 mL) was collected after the prostatic massage, as previously described by Lewis *et al*.^20^. The samples were collected using Urine Preservative (Norgen, Biotek Corp, Ontario, Canada), which is designed for the preservation of nucleic acids and proteins in fresh urine samples at room temperature. Urine was centrifuged at 650 g × 5 min. Once the supernatant was discarded, the urine sediment was washed twice with phosphate-buffered saline (PBS) and RNA extraction was performed using Urine Exfoliated Cell and Bacteria RNA Purification Kit (Norgen, Biotek Corp, Ontario, Canada) slightly modifying manufacturers’ instructions. To remove genomic DNA, all the samples were treated with RNase-free DNase I according to the manufacturer’s protocol (Norgen, Biotek Corp, Ontario, Canada). RNA concentration was assessed using the Qubit RNA assay (ThermoFisher Scientific, Kandel, Germany) and it was stored at −80 °C until further investigation.

### Small RNA sequencing

Indexed libraries of PCa patients were prepared from purified RNA (25 ng) with SMARTer® smRNA-Seq Kit for Sample Prep Kit (Clontech Laboratories, Mountain View, CA, USA) according to manufacturer’s instructions. Libraries were quantified using the Tape Station 4200 (Agilent Technologies, Santa Clara, CA, USA) and pooled to obtain an index-tagged sample present in equimolar amounts, with a final concentration of the pooled samples of 2 nM. The pooled samples were subject to cluster generation and sequencing using an Illumina HiSeq2500 System (Illumina, San Diego, CA, USA) in a 1 × 50 single read format with a final concentration of 10 pM.

### Data processing miRNA sequencing

The raw sequences file generated underwent quality control analysis using FASTQC (http://www.bioinformatics.babraham.ac.uk/projects/fastqc/). Before aligning sequences to a reference genome, the sequencing adaptors (Illumina), which were used in the sequencing library preparation, were trimmed using sRNAtoolbox. In order to generate sRNAbench sequence alignments, sRNAtoolbox was used. The alignment was performed setting the following parameters: seed length for alignment = 20, minimum read-count = 2, bowtie-alignment type = n, allowed number of mismatch = 0, minimum read length = 15 and maximum number of multiple mappings = 10. MiRNAs expression among the several samples was normalized to Read per Million (RPM) value, as computed by sRNAbench. Heat maps of expressed miRNAs were generated using tMev version 4.9 on RPM normalized values.

### Prediction of miRNA targets and functional analysis of target genes

Computationally predicted mRNA targets of the top-62 miRNAs were identified using “microRNA Target Filter” of Ingenuity Pathway Analysis (IPA); considering TarBase mRNA target^21^. We performed a Gene Ontology (GO) and pathways analysis in order to investigate the relationships between these genes and PCa. IPA “Core Analysis” was used and only statistically significant data was considered (FDR ≤ 0.05, Benjamini-Hochberg for “Biological Function” and P ≤ 0.05 for pathways analysis).

### Let-7 family qPCR in PCa and in age-matched healthy control subjects

The urine samples were collected using Urine Preservative (Norgen, Biotek Corp, Ontario, Canada) and Urine Exfoliated Cell and Bacteria RNA Purification Kit (Norgen, Biotek Corp, Ontario, Canada) was used to isolate RNA in 11 healthy subjects (control). After extraction, RNA was subjected to qualitative assessment through Bioanalyzer and NanoDrop. Total RNA was quantified by Qubit RNA Assay (ThermoFisher Scientific, Kandel, Germany) and stored at −80 °C until use. RNA used for 11 PCa patients previously extracted for library purified preparation (as previously described). Purified RNA (10 ng) derived from C and PCa subjects was reverse-transcribed using cDNA Universal cDNA Synthesis Kit II (Exiqon, Vedbæk, Denmark), according to manufacturer’s protocol. RNA spike-in control (0.5 µL) was introduced in 10 µL total volume of the retrotranscription mix before incubation as positive cDNA synthesis control (UniSp6, provided with cDNA synthesis kit). The procedure was optimized to maximize signal and minimize effects of inhibitors as recommended by Instruction Manual for miRCURY LNA™ Universal RT microRNA PCR.

QPCR amplification was carried out using 4 µL of cDNA (diluted 1:20), 5 µL of ExiLENT SYBR® Green master mix (Exiqon, Vedbæk, Denmark) and 1 µL of miRCURY LNA specific PCR primer (Exiqon, Vedbæk, Denmark) in a final volume of 10 μL according to manufacturer’s recommendations.

The following microRNA LNA™ PCR primers (Exiqon, Vedbæk, Denmark) were used for qPCR: hsa-let-7a-5p (Exiqon product number 205727), hsa-let-7b-5p (204750), hsa-let-7c-5p (204767), hsa-let-7d-5p (204124), hsa-let-7e-5p (205711), hsa-let-7f-5p (204359), hsa-let-7g-5p (204565) and hsa-let-7i-5p (204394).

All quantitative PCR values were normalized to has-miR-191 (Exiqon product number 204306) reference gene, in accordance with previously published paper^14^. Thermo-cycling conditions on iCycler iQ Real-Time PCR (Bio-Rad, Hercules, CA) consisted of an initial denaturation of 10 min at 95 °C, followed by 45 cycles of 15 s at 95 °C for denaturing double stranded DNA and 1 min at 60 °C for annealing/extension steps. Melting curves were plotted at the end of each cycle series to assess amplification specificity, through the verification of the presence of one-gene specific peak and the absence of primer dimers. Three biological replicates were used for the qPCR to verify the expression level of miRNAs and no template controls (NTC) were included in the analysis. For each sample, LNA control primer set was used to amplify UniSp4 and UniSp6 spike-in positive control. Bio-Rad software plotted the fluorescence intensity against the number of cycles providing cycle threshold (Ct) value using the automatic method.

To normalize expression value of target miRNAs, 2^-ΔCt^ method was used (ΔCt = Ct target miRNA - Ct has-miR-191)^22,23^.

### Statistical analysis of Let-7 family qPCR data

RT-qPCR technical replicates were averaged and only samples with a standard error lower than 0.2 C_t_ were maintained. The dataset normality was tested by Shapiro-Wilks test and a P ≤ 0.05 was considered statistical significant. T-test for independent samples was used. Statistical analysis was carried out with R software.

## Results

### RNA quality and quantity

Mean RNA integrity number (RIN) was 8.7 (range 8.3–9.0) and 260/280 ratio was 1.84 (range 1.84–2.03), indicating the high purity of RNA preparation in every sample. In healthy men RNA yield was approximately 10 ng/µL (0.5 µg RNA/30 mL urine) and 50 ng/µL (2.5 µg RNA/30 mL urine) in PCa subjects.

### NGS of PCa miRNA

**Figure 1:**
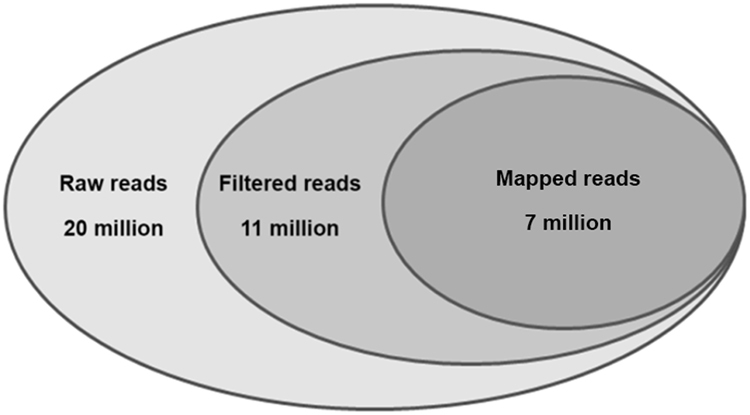
Sequencing results. Deep sequencing results overview showing the average number of raw reads, filtered reads and mapped reads obtained in the small-RNA sequencing experiment. Filtered and mapped reads were analyzed using sRNABench bioinformatics tool.

MiRNA profile was examined using Illumina small RNA sequencing. For all PCa samples, in average 20071425 raw reads were obtained. After trimming the adapter sequence and removing those with low quality, we obtained, in average, 11275265 reads and of these, about 60% can be mapped to the human genome (hg19) with high confidence (Fig. 1). MiRNA was considered expressed when detected, in at least one sample, with a RPM normalized expression value ≥ the first quantile of expression distribution. Applying this statistical cut-off, we identified 236 miRNAs as shown in supplemental table.

### Gene ontology analysis and Pathway analysis of the predicted miRNA target genes

To predict mRNA targets and biological pathways, we selected the top-62 expressed miRNAs including *let-7* family validated by qPCR assay.

**Figure 2:**
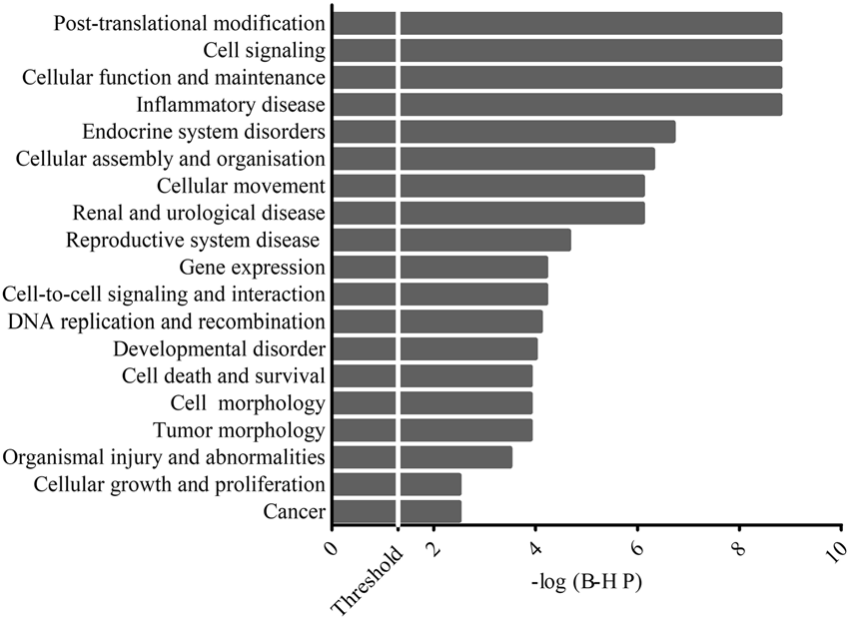
List of diseases and functions of miRNAs target. Bar-chart showing the 19 Gene ontologies found to be most highly statistically enriched in response to PCa urine cells miRNAs (FDR ≤ 0.05 Benjamini-Hochberg). MiRNA targets were computed using “microRNA Target Filter” of Ingenuity Pathway Analysis, taking in consideration only validated mRNAs target listed in TarBase.

**Figure 3:**
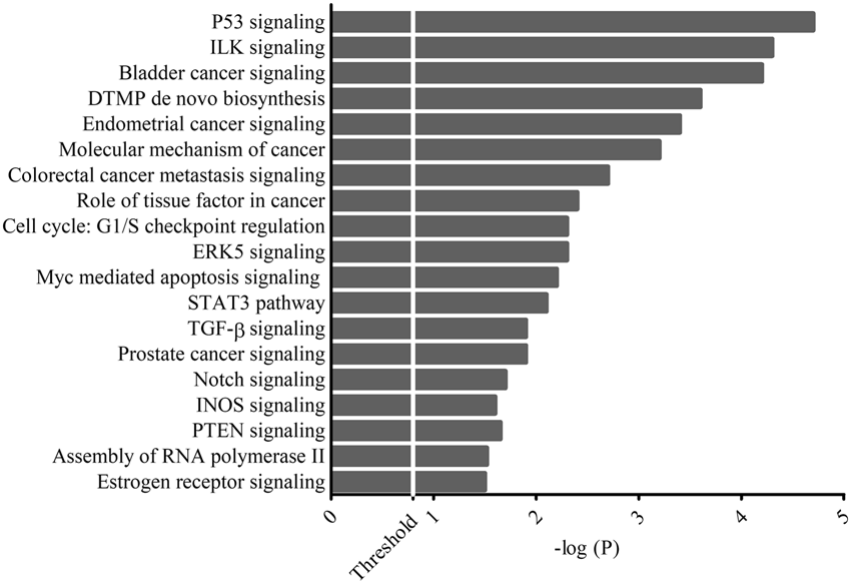
List of statistically enriched pathways of miRNAs target. Bar-chart showing the statistically enriched pathways (P ≤ 0.05, Fisher’s test) where the mRNAs target of miRNAs are involved. Pathways analysis was performed using “Core Analysis” function of Ingenuity Pathway Analysis software.

Functional analysis was performed with Core Analysis and revealed that most top-62 were involved in cancer, cellular development, cellular growth and proliferation and cell death and cell survival (FDR ≤ 0.05, Benjamini-Hochberg) (Fig. 2). Interestingly, among the several pathways statistically enriched (P ≤ 0.05, Fisher’s test), some genes were involved in Prostate Cancer Signaling, especially *Ras*, *P ten* and *Cyclin D1* (Fig. 3).

### QPCR validation of let-7 family

**Figure 4:**
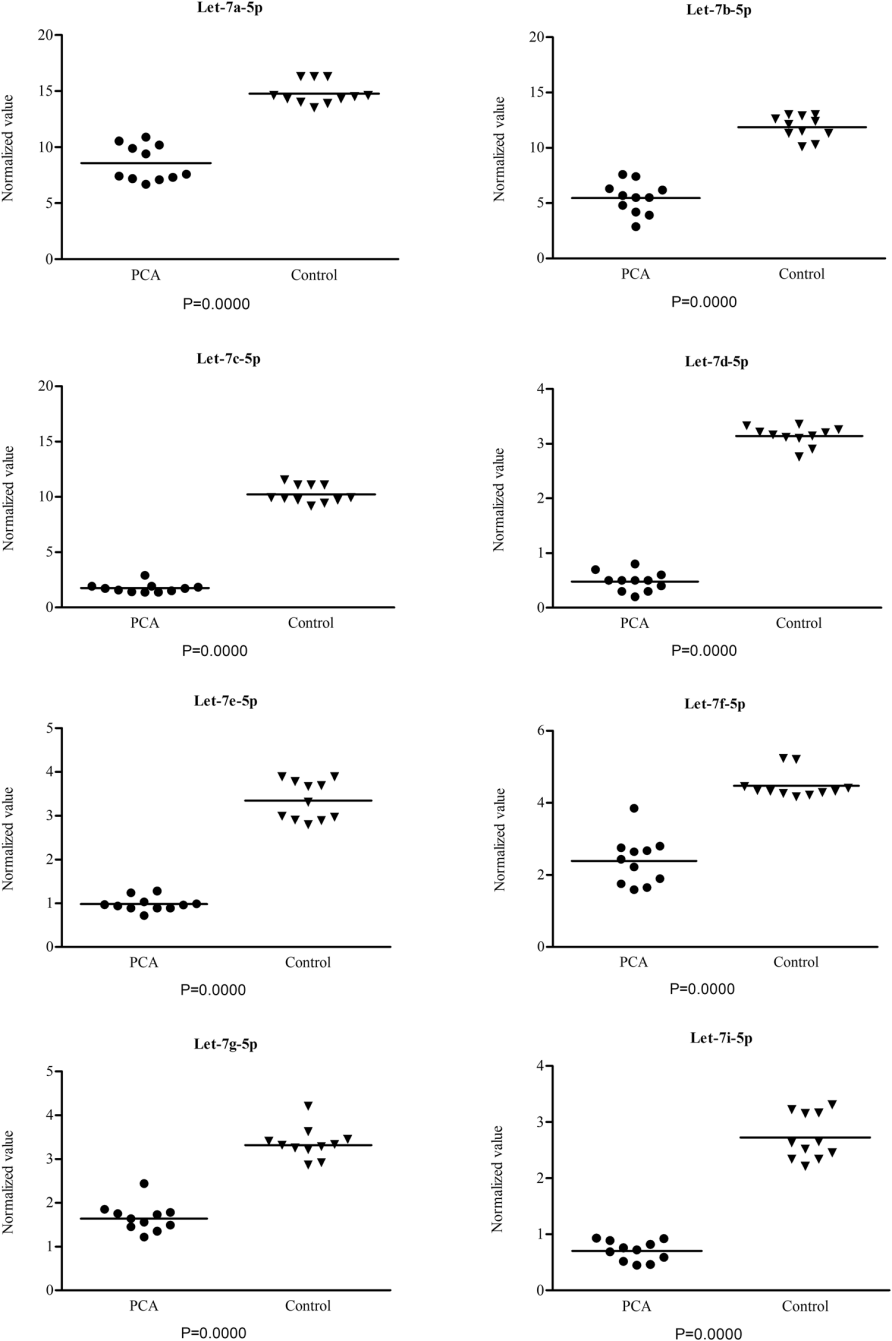
MiRNA expression levels. Normalized expression value (2^-ΔCt^) of *let-7* family miRNAs in post-DRE urine sediments from PCa (left) and controls subjects. The solid symbols show the observed subjects and the bar indicate the mean of observations. The 2^-ΔCt^ method was used to calculate the miRNAs normalized expression as follows: 2^-(Ct target miRNA - Ct miR-191)^.

To validate the activities of PCa miRNAs predicted by NGS we selected let-7 family. Let-7 family were amplified by qPCR in age-matched healthy control and PCa subjects. *Let-7* family miRNA panel was chosen for the potential promising diagnostic and therapeutic role in PCa. In fact, results showed that PCa *let-7* family was down-regulated (P ≤ 0.0001) compared to healthy controls, as shown in Fig. 4.

## Discussion

Given the indolent nature of localized low-grade PCa, its over detection has led to overtreatment in many affected men. The identification of molecular biomarkers has been researched thoroughly in order to identify PCa early and robustly. *De facto*, PSA-based screening is limited in that a non-trascurable amount of high-risk cancer will not be detectable. Since 1997 the European Randomised Study of Screening for Prostate Cancer (ERSPC) set the PSA > 3 ng/mL as an indication to prostate biopsy overlooking other imaging or clinical aspects^6,24–29^.

The widespread diffusion of mpMRI has changed the diagnostic paradigm of localized PCa. This imaging modality has a high sensitivity and high negative predictive value for the aggressive disease^30^. To improve PCa detection rate, biopsy of the suspicious area should be performed after mp-MRI of the prostate, even using a simple cognitive fusion technique, based on the observation and localization of prostatic lesions on the MRI image. At least 12 cores should be taken in each patient, in consideration of multifocal entity of PCa^31^.

The development of a panel of biomarkers could be helpful in diagnostic and prognostic accuracy. The number of potential biomarkers in PCa will rise in the next years. In particular, miRNAs are object of intense study^5,32,33^, as they can be extracted from a wide range of biological samples and are highly stable in various storage conditions^34^. In this study, we investigated UEPC miRNA expression levels using NGS technology to identify the entire miRNome in PCa patients. Subsequently, we compared levels of the highest selected PCa miRNAs with those in age-matched healthy control subjects. The main advantages of UEPC collection is a non-invasive and relatively fast and cost-efficient procedure compared with other clinical samples^18^. Due to the anatomic proximity of the prostate gland to the urethra, detection of biomarkers may be found in urine. In particular, when the sample is enriched with prostate cells after performing a prostate massage^35^. Urine after DRE is the only biological matrix containing prostate cancer-derived cells, furthermore, this non-invasive sampling permits repeated procedures and follow-up monitoring.

The first study on altered miRNAs in PCa began in 2007 and aimed to identify a specific panel by systematically profiling prostate cancer cell lines^36^. Since then, several authors proposed different miRNA profiles, using different approaches^37^. To the best of our knowledge, this research is among the first to utilize NGS Illumina Deep Sequencing to examine the entire spectrum of miRNAs from UEPCs. The key advantage of small RNA sequencing is the identification of low miRNA abundance and the successful detection of novel miRNAs without *a priori* knowledge of sequences^9^.

Through the NGS approach we performed the GO analysis of top-60 miRNAs, allowing us an enrichment analysis by comparing top-60 miRNAs to all available biological pathways. Our study provides additional evidence that top-60 miRNAs are involved in Prostate Cancer Signaling, in particular, through the modulation of *Ras*, *P ten* and *Cyclin D1* genes. One suggested function of *Pten* in the nucleus is to induce G1 cell cycle arrest partially by the reduction of *Cyclin D1* levels through its protein phosphatase activity^38^ or through controlling MAPK signaling^39^. Recent research using NGS suggests *Ras/MAPK* as a potential driver in human prostate cancer pathogenesis and progression^40,41^. We have validated *let-7* family among all miRNAs for their potential promising therapeutic role in PCa. It showed the highest AVG Expression RPM and in one of our previous papers^15^, demonstrated that *let-7* family was able to discriminate in serum patients with PCa from those harboring BPH when both presented altered PSA levels (3 ng/mL)^15^.

Originally, the *let-7* family was discovered as a crucial gene in *Caenorhabditis elegans* and, later, as one of the first miRNAs^42^. Even if several characteristics of *let-7* family are conserved across species, there are some remarkable differences between the *let-7* family genes in *C. elegans* and orthologous genes in more complex organisms. For instance, in humans, thirteen *let-7* family precursor miRNAs were described and coded 10 different mature *let-7* miRNAs isoform^43^. Although the role of this miRNA family is not fully understood, its aberrant expression is associated with cancer^44,45^.

The results obtained in our previous research^15^ are in accordance with our findings. In conclusion, our results lead us to hypothesize that the profile of let-7 family in UEPC could really become new, easily accessible, affordable, non-invasive, and promising testing tool for the personalized management of patients with PCa. Probably, during the next few years, the circulating let-7 expression analysis may be a useful tool to predict the therapeutic outcomes of patients’ response to specific disease states and oligonucleotide antagonists therapy with let-7 miRNA-inhibitor will become available to determine the therapeutic future of this potentially very powerful technology.

## Electronic supplementary material

List of miRNAs identified in tumoral samples

## References

[1] Dall’Era MA (2012). Active surveillance for prostate cancer: a systematic review of the literature. Eur. Urol..

[2] Xu W, Zhou M (2014). A concise update on prostate pathology. Českoslov. Patol..

[3] Loeb S (2013). Systematic review of complications of prostate biopsy. Eur. Urol..

[4] Wittmann J, Jäck H-M (2010). Serum microRNAs as powerful cancer biomarkers. Biochim. Biophys. Acta.

[5] Fabris L (2016). The Potential of MicroRNAs as Prostate Cancer Biomarkers. Eur. Urol..

[6] Van Neste, L. *et al*. Detection of High-grade Prostate Cancer Using a Urinary Molecular Biomarker-Based Risk Score. *Eur. Urol*. 10.1016/j.eururo.2016.04.012 (2016). 10.1016/j.eururo.2016.04.012 27108162

[7] Ambros V (2004). The functions of animal microRNAs. Nature.

[8] Giraldez AJ (2006). Zebrafish MiR-430 promotes deadenylation and clearance of maternal mRNAs. Science.

[9] Song C (2015). Expression profile analysis of microRNAs in prostate cancer by next-generation sequencing. The Prostate.

[10] Inui M, Martello G, Piccolo S (2010). MicroRNA control of signal transduction. Nat. Rev. Mol. Cell Biol..

[11] Fang Y-X, Gao W-Q (2014). Roles of microRNAs during prostatic tumorigenesis and tumor progression. Oncogene.

[12] Masood N, Yasmin A (2017). Entangling Relation of Micro RNA-let7, miRNA-200 and miRNA-125 with Various Cancers. Pathol. Oncol. Res. POR.

[13] Egidi, M. G. *et al*. Characterization of Kallireins and microRNAs in Urine Sediment for the Discrimination of Prostate Cancer from Benign Prostatic Hyperplasia. *J. Cancer Sci. Ther*. **7** (2015).

[14] Egidi MG (2015). Stability Assessment of Candidate Reference Genes in Urine Sediment of Prostate Cancer Patients for miRNA Applications. Dis. Markers.

[15] Cochetti G (2016). Different levels of serum microRNAs in prostate cancer and benign prostatic hyperplasia: evaluation of potential diagnostic and prognostic role. OncoTargets Ther..

[16] Creighton CJ, Reid JG, Gunaratne PH (2009). Expression profiling of microRNAs by deep sequencing. Brief. Bioinform..

[17] Tam S, de Borja R, Tsao M-S, McPherson JD (2014). Robust global microRNA expression profiling using next-generation sequencing technologies. Lab. Investig. J. Tech. Methods Pathol..

[18] Matullo G, Naccarati A, Pardini B (2016). MicroRNA expression profiling in bladder cancer: the challenge of next-generation sequencing in tissues and biofluids. Int. J. Cancer.

[19] Mestdagh P (2014). Evaluation of quantitative miRNA expression platforms in the microRNA quality control (miRQC) study. Nat. Methods.

[20] Lewis H (2014). miR-888 is an expressed prostatic secretions-derived microRNA that promotes prostate cell growth and migration. Cell Cycle Georget. Tex.

[21] Sethupathy P, Corda B, Hatzigeorgiou AG (2006). TarBase: A comprehensive database of experimentally supported animal microRNA targets. RNA N. Y. N.

[22] Schmittgen TD, Livak KJ (2008). Analyzing real-time PCR data by the comparative C(T) method. Nat. Protoc..

[23] Livak KJ, Schmittgen TD (2001). Analysis of relative gene expression data using real-time quantitative PCR and the 2(-Delta Delta C(T)) Method. Methods San Diego Calif.

[24] Bill-Axelson A (2014). Radical prostatectomy or watchful waiting in early prostate cancer. N. Engl. J. Med..

[25] Schröder, F. H., Raaijmakers, R., Postma, R., van der Kwast, T. H. & Roobol, M. J. 4-year prostate specific antigen progression and diagnosis of prostate cancer in the European Randomized Study of Screening for Prostate Cancer, section Rotterdam. *J. Urol*. **174**, 489–494; discussion 493–494 (2005). 10.1097/01.ju.0000165568.76908.5c 16006878

[26] Postma R, Schröder FH (2005). Screening for prostate cancer. Eur. J. Cancer Oxf. Engl..

[27] Postma R (2005). Incidence and follow-up of patients with focal prostate carcinoma in 2 screening rounds after an interval of 4 years. Cancer.

[28] Catalona WJ (1994). Screening for prostate cancer. Lancet Lond. Engl..

[29] Catalona WJ (1994). Comparison of digital rectal examination and serum prostate specific antigen in the early detection of prostate cancer: results of a multicenter clinical trial of 6,630 men. J. Urol..

[30] Fütterer JJ (2015). Can Clinically Significant Prostate Cancer Be Detected with Multiparametric Magnetic Resonance Imaging? A Systematic Review of the Literature. Eur. Urol..

[31] Fandella A (2017). Italian Prostate Biopsies Group: 2016 Updated Guidelines Insights. Anticancer Res..

[32] Catto JWF (2011). MicroRNA in prostate, bladder, and kidney cancer: a systematic review. Eur. Urol..

[33] Ferro M (2016). Biomarkers in localized prostate cancer. Future Oncol. Lond. Engl..

[34] Chen X (2008). Characterization of microRNAs in serum: a novel class of biomarkers for diagnosis of cancer and other diseases. Cell Res..

[35] Filella, X. & Foj, L. Prostate Cancer Detection and Prognosis: From Prostate Specific Antigen (PSA) to Exosomal Biomarkers. *Int. J. Mol. Sci*. **17** (2016). 10.3390/ijms17111784 PMC5133785 27792187

[36] Porkka KP (2007). MicroRNA expression profiling in prostate cancer. Cancer Res..

[37] Bertoli G, Cava C, Castiglioni I (2016). MicroRNAs as Biomarkers for Diagnosis, Prognosis and Theranostics in Prostate Cancer. Int. J. Mol. Sci..

[38] Weng, L. P., Brown, J. L. & Eng, C. PTEN coordinates G(1) arrest by down-regulating cyclin D1 via its protein phosphatase activity and up-regulating p27 via its lipid phosphatase activity in a breast cancer model. *Hum. Mol. Genet*. **10**, 599–604 (2001). 10.1093/hmg/10.6.599 11230179

[39] Chung J-H (2006). The ERK1/2 pathway modulates nuclear PTEN-mediated cell cycle arrest by cyclin D1 transcriptional regulation. Hum. Mol. Genet..

[40] Bakin RE, Gioeli D, Sikes RA, Bissonette EA, Weber MJ (2003). Constitutive activation of the Ras/mitogen-activated protein kinase signaling pathway promotes androgen hypersensitivity in LNCaP prostate cancer cells. Cancer Res..

[41] Colombatti M (2009). The prostate specific membrane antigen regulates the expression of IL-6 and CCL5 in prostate tumour cells by activating the MAPK pathways. PloS One.

[42] Reinhart BJ (2000). The 21-nucleotide let-7 RNA regulates developmental timing in Caenorhabditis elegans. Nature.

[43] Roush S, Slack FJ (2008). The let-7 family of microRNAs. Trends Cell Biol..

[44] Johnson CD (2007). The let-7 microRNA represses cell proliferation pathways in human cells. Cancer Res..

[45] Boyerinas B, Park S-M, Hau A, Murmann AE, Peter ME (2010). The role of let-7 in cell differentiation and cancer. Endocr. Relat. Cancer.

