# Supplementary material for: Next Generation Sequencing of urine exfoliated cells: an approach of prostate cancer microRNAs research
Source: Sci Rep. 2018 May 8;8:7111. doi: 10.1038/s41598-018-24236-y (PMC5940782; doi:10.1038/s41598-018-24236-y)
Supplement: Supplementary file 1 — List of miRNAs identified in tumoral samples [file 41598_2018_24236_MOESM1_ESM.pdf]

Next Generation Sequencing of urine exfoliated cells: an approach of prostate cancer microRNAs research

Guelfi Gabriella[1], Cochetti Giovanni[2], Stefanetti Valentina[1], Zampini Danilo[1], Diverio Silvana[1], Boni Andrea[2], Mearini Ettore[2]

Supplementary Table
List of miRNAs identified in tumoral samples. A miRNA is considered expressed if it has a normalized expression value ≥ I quantile of expression distribution in at least one sample

Number	MiRNA Name	AVG Expression RPM
**1**	**hsa-let-7a-5p**	**594,70**
2	hsa-miR-6087	522,65
**3**	**hsa-let-7b-5p**	**463,53**
4	hsa-miR-4516	410,34
5	hsa-miR-4532	355,66
6	hsa-miR-223-3p	261,10
7	hsa-miR-4488	204,76
8	hsa-miR-7704	183,89
9	hsa-miR-203a-3p	149,13
10	hsa-miR-24-3p	146,86
**11**	**hsa-let-7f-5p**	**143,10**
12	hsa-miR-19b-3p	133,43
**13**	**hsa-let-7g-5p**	**112,58**
14	hsa-miR-4492	109,04
15	hsa-miR-191-5p	103,87
**16**	**hsa-let-7c-5p**	**99,93**
17	hsa-miR-1246	87,64
18	hsa-miR-25-3p	81,92
19	hsa-miR-23a-3p	73,13
20	hsa-miR-205-5p	70,61
21	hsa-miR-29a-3p	68,77
22	hsa-miR-29c-3p	64,97
23	hsa-miR-21-5p	61,46
24	hsa-miR-125a-5p	47,40
25	hsa-miR-23b-3p	46,04
26	hsa-miR-29b-3p	45,79
27	hsa-miR-5100	42,44
28	hsa-miR-200c-3p	41,40
29	hsa-miR-92a-3p	39,25
30	hsa-miR-3656	37,10
31	hsa-miR-4485-5p	36,50

32	hsa-miR-125b-5p	35,89
33	hsa-miR-423-5p	35,65
34	hsa-miR-141-3p	34,29
35	hsa-miR-574-3p	29,29
36	hsa-miR-342-3p	24,29
37	hsa-miR-4485-3p	23,87
38	hsa-miR-93-5p	22,97
**39**	**hsa-let-7e-5p**	**21,76**
40	hsa-miR-30e-3p	21,75
41	hsa-miR-146a-5p	21,28
42	hsa-miR-30c-5p	20,49
43	hsa-miR-4284	19,53
44	hsa-miR-19a-3p	18,65
45	hsa-miR-200b-3p	17,56
46	hsa-miR-142-5p	16,81
47	hsa-miR-30b-5p	14,77
48	hsa-miR-30a-3p	14,52
49	hsa-miR-375	14,51
50	hsa-miR-26a-5p	14,24
51	hsa-miR-186-5p	13,94
52	hsa-miR-28-3p	13,63
53	hsa-miR-148a-3p	13,47
54	hsa-miR-429	13,35
55	hsa-miR-20b-5p	12,54
56	hsa-miR-22-3p	12,39
**57**	**hsa-let-7i-5p**	**12,30**
58	**hsa-let-7d-5p**	11,91
59	hsa-miR-135a-5p	11,72
60	hsa-miR-26b-5p	11,33
61	hsa-miR-27a-3p	11,09
62	hsa-miR-335-5p	10,91
63	hsa-miR-200a-3p	10,74
64	hsa-miR-363-3p	10,16
65	hsa-miR-200b-5p	10,10
66	hsa-miR-151a-3p	10,04
67	hsa-miR-1260b	9,90
68	hsa-miR-424-5p	9,56
69	hsa-miR-149-5p	9,29
70	hsa-miR-101-3p	9,17
71	hsa-miR-192-5p	9,04
72	hsa-miR-16-5p	8,51
73	hsa-miR-17-5p	7,28
74	hsa-miR-194-5p	6,97
75	hsa-miR-98-5p	6,80
76	hsa-miR-374b-5p	6,51
77	hsa-miR-27b-3p	6,37

78	hsa-miR-423-3p	6,15
79	hsa-miR-660-5p	6,02
80	hsa-miR-221-3p	5,97
81	hsa-miR-31-5p	5,92
82	hsa-miR-210-3p	5,78
83	hsa-miR-4508	5,70
84	hsa-miR-6126	5,41
85	hsa-miR-3195	5,35
86	hsa-miR-3196	5,15
87	hsa-miR-151a-5p	5,09
88	hsa-miR-4497	4,92
89	hsa-miR-320a	4,75
90	hsa-miR-7-1-3p	4,63
91	hsa-miR-378a-3p	4,56
92	hsa-miR-196b-5p	4,53
93	hsa-miR-99a-5p	4,45
94	hsa-miR-224-5p	4,42
95	hsa-miR-151b	4,14
96	hsa-let-7d-3p	4,06
97	hsa-miR-4792	4,01
98	hsa-miR-1260a	3,79
99	hsa-miR-34a-5p	3,62
100	hsa-miR-28-5p	3,56
101	hsa-miR-142-3p	3,29
102	hsa-miR-143-3p	3,28
103	hsa-miR-222-3p	3,03
104	hsa-miR-30d-5p	2,97
105	hsa-miR-103a-3p	2,89
106	hsa-miR-331-3p	2,78
107	hsa-miR-146b-5p	2,68
108	hsa-miR-328-3p	2,55
109	hsa-miR-425-5p	2,54
110	hsa-miR-532-3p	2,49
111	hsa-miR-99b-5p	2,49
112	hsa-let-7b-3p	2,48
113	hsa-miR-532-5p	2,36
114	hsa-miR-3178	2,27
115	hsa-miR-10b-5p	2,27
116	hsa-miR-150-5p	2,26
117	hsa-miR-4510	2,22
118	hsa-miR-185-5p	2,14
119	hsa-let-7a-3p	2,08
120	hsa-miR-374a-3p	2,06
121	hsa-miR-324-5p	1,99
122	hsa-miR-484	1,98
123	hsa-miR-4454	1,97

124	hsa-miR-374a-5p	1,92
125	hsa-miR-193a-5p	1,92
126	hsa-miR-140-5p	1,66
127	hsa-miR-148b-3p	1,64
128	hsa-miR-486-5p	1,54
129	hsa-miR-30e-5p	1,45
130	hsa-miR-30a-5p	1,44
131	hsa-miR-4443	1,38
132	hsa-miR-22-5p	1,34
133	hsa-miR-7977	1,33
134	hsa-miR-590-5p	1,27
135	hsa-miR-744-5p	1,25
136	hsa-miR-664a-3p	1,22
137	hsa-miR-223-5p	1,20
138	hsa-miR-29c-5p	1,20
139	hsa-miR-1275	1,19
140	hsa-miR-663a	1,18
141	hsa-miR-140-3p	1,18
142	hsa-miR-7-5p	1,12
143	hsa-miR-361-5p	1,10
144	hsa-miR-200a-5p	1,07
145	hsa-miR-361-3p	0,94
146	hsa-miR-183-5p	0,91
147	hsa-miR-505-3p	0,90
148	hsa-miR-106a-3p	0,84
149	hsa-miR-324-3p	0,83
150	hsa-miR-590-3p	0,79
151	hsa-miR-20a-5p	0,78
152	hsa-miR-33a-3p	0,78
153	hsa-miR-3065-5p	0,75
154	hsa-miR-106a-5p	0,74
155	hsa-miR-15b-5p	0,72
156	hsa-miR-338-3p	0,72
157	hsa-miR-195-5p	0,72
158	hsa-miR-4417	0,71
159	hsa-miR-130a-3p	0,68
160	hsa-miR-451a	0,67
161	hsa-miR-497-5p	0,61
162	hsa-miR-598-3p	0,60
163	hsa-miR-107	0,60
164	hsa-miR-126-5p	0,59
165	hsa-miR-15b-3p	0,59
166	hsa-miR-193b-3p	0,58
167	hsa-miR-141-5p	0,55
168	hsa-miR-199b-5p	0,55
169	hsa-miR-345-5p	0,53

170	hsa-miR-130b-3p	0,52
171	hsa-miR-301a-3p	0,52
172	hsa-miR-32-5p	0,52
173	hsa-miR-708-5p	0,51
174	hsa-miR-320b	0,49
175	hsa-miR-3960	0,49
176	hsa-miR-326	0,48
177	hsa-miR-452-5p	0,48
178	hsa-miR-126-3p	0,47
179	hsa-miR-21-3p	0,47
180	hsa-miR-29a-5p	0,46
181	hsa-miR-30d-3p	0,46
182	hsa-miR-4449	0,45
183	hsa-miR-874-3p	0,45
184	hsa-miR-4461	0,45
185	hsa-miR-93-3p	0,44
186	hsa-miR-664b-3p	0,42
187	hsa-miR-128-3p	0,41
188	hsa-miR-32-3p	0,40
189	hsa-miR-92b-3p	0,40
190	hsa-miR-181a-5p	0,40
191	hsa-miR-425-3p	0,40
192	hsa-miR-1973	0,39
193	hsa-miR-204-5p	0,39
194	hsa-miR-424-3p	0,37
195	hsa-miR-199a-3p=hsa-miR-199b-3p	0,37
196	hsa-miR-378a-5p	0,36
197	hsa-miR-3135b	0,33
198	hsa-miR-362-3p	0,32
199	hsa-miR-454-3p	0,32
200	hsa-miR-193b-5p	0,32
201	hsa-miR-20a-3p	0,32
202	hsa-miR-145-5p	0,31
203	hsa-miR-378c	0,31
204	hsa-miR-15a-5p	0,29
205	hsa-miR-152-3p	0,29
206	hsa-miR-340-5p	0,29
207	hsa-miR-1307-3p	0,28
208	hsa-miR-6773-5p	0,28
209	hsa-miR-4484	0,28
210	hsa-miR-3607-3p	0,28
211	hsa-miR-30b-3p	0,28
212	hsa-miR-190a-5p	0,27
213	hsa-miR-106b-5p	0,27
214	hsa-miR-215-5p	0,26
215	hsa-miR-542-3p	0,25

216	hsa-miR-3613-5p	0,24
217	hsa-miR-5701	0,22
218	hsa-miR-3687	0,21
219	hsa-miR-26b-3p	0,21
220	hsa-miR-153-3p	0,20
221	hsa-miR-132-3p	0,20
222	hsa-miR-658	0,20
223	hsa-miR-95-3p	0,19
224	hsa-miR-877-5p	0,17
225	hsa-miR-374c-5p	0,17
226	hsa-miR-31-3p	0,17
227	hsa-miR-3653-3p	0,16
228	hsa-miR-10a-5p	0,16
229	hsa-miR-628-3p	0,16
230	hsa-miR-363-5p	0,14
231	hsa-miR-664a-5p	0,14
232	hsa-miR-195-3p	0,14
233	hsa-miR-6798-5p	0,13
234	hsa-miR-144-5p	0,12
235	hsa-miR-147b	0,11
236	hsa-miR-663b	0,11
